# Effect of Six-Month Diet Intervention on Sleep among Overweight and Obese Men with Chronic Insomnia Symptoms: A Randomized Controlled Trial

**DOI:** 10.3390/nu8110751

**Published:** 2016-11-23

**Authors:** Xiao Tan, Markku Alén, Kun Wang, Jarkko Tenhunen, Petri Wiklund, Markku Partinen, Sulin Cheng

**Affiliations:** 1 Exercise Health and Technology Center, Shanghai Jiao Tong University, Shanghai 200240, China; xiao.tan@jyu.fi (X.T.); wangkunz@sjtu.edu.cn (K.W.); petri.wiklund@jyu.fi (P.W.); 2 Department of Health Sciences, University of Jyväskylä, Jyväskylä 40014, Finland; markku.alen@jyu.fi (M.A.); tenhujar@gmail.com (J.T.); 3 Department of Medical Rehabilitation, Oulu University Hospital and Center for Life Course Health Research, University of Oulu, Oulu 90220, Finland; 4 VitalMed Research Center, Helsinki Sleep Clinic and Department of Neurosciences, University of Helsinki, Helsinki 00380, Finland; markku.partinen@helsinki.fi

**Keywords:** insomnia symptoms, sleep, sleep onset, diet intervention, nutrient, overweight, obesity

## Abstract

Growing evidence suggests that diet alteration affects sleep, but this has not yet been studied in adults with insomnia symptoms. We aimed to determine the effect of a six-month diet intervention on sleep among overweight and obese (Body mass index, BMI ≥ 25 kg/m^2^) men with chronic insomnia symptoms. Forty-nine men aged 30–65 years with chronic insomnia symptoms were randomized into diet (*n* = 28) or control (*n* = 21) groups. The diet group underwent a six-month individualized diet intervention with three face-to-face counseling sessions and online supervision 1–3 times per week; 300–500 kcal/day less energy intake and optimized nutrient composition were recommended. Controls were instructed to maintain their habitual lifestyle. Sleep parameters were determined by piezoelectric bed sensors, a sleep diary, and a Basic Nordic sleep questionnaire. Compared to the controls, the diet group had shorter objective sleep onset latency after intervention. Within the diet group, prolonged objective total sleep time, improved objective sleep efficiency, lower depression score, less subjective nocturnal awakenings, and nocturia were found after intervention. In conclusion, modest energy restriction and optimized nutrient composition shorten sleep onset latency in overweight and obese men with insomnia symptoms.

## 1. Introduction

Insomnia is a highly prevalent sleep disorder and has become a significant health issue in many countries. The prevalence of chronic insomnia symptoms classified by the Diagnostic and Statistical Manual of Mental Disorders, 4th Edition (DSM-IV) criteria ranges between 15.2% and 22.1% of the general population in different regions of the world [1,2,3]. In Finland, nearly a quarter of the employed people are reported to suffer from insomnia [4]. Insomnia symptoms are risk factors of various adverse health consequences [5]. For instance, difficulty initiating sleep, the symptom represented by prolonged sleep onset latency (SOL), is independently associated with all-cause mortality among Finnish men [6].

A growing body of evidence suggests that overweight and obesity are significant risk factors for impaired sleep and insomnia [7,8,9]. Population-based study showed that obese adults had higher incidence of subjective sleep disturbances than non-obese ones [9]. Longitudinal studies suggested that both obesity and weight gain could predict future development of insomnia symptoms [7,8]. Diet is an important mediator between sleep and overweight/obesity. Growing evidence suggest that diet alterations can directly influence sleep parameters [10,11]. Furthermore, the associations between nutrients and insomnia symptoms have been reported by recent studies [12,13,14]. However, no previous study has investigated whether diet intervention leads to improved sleep parameters related to insomnia symptoms, such as sleep onset latency. There is also a lack of data regarding the effects of diet-induced weight loss on sleep among overweight and obese populations, especially in men, among which the combined prevalence of overweight and obesity is higher than women in Finland [15].

Thus, the present study aimed to investigate whether sleep parameters among overweight and obese men with chronic insomnia symptoms can be improved through a six-month diet intervention. We hypothesized that reduced energy intake and optimized nutrient composition can improve one or multiple objectively and subjectively measured sleep parameters.

## 2. Methods

The present randomized controlled trial forms part of a larger study with different lifestyle interventions on middle-aged men with sleep disorders (Monitoring and treatment of obesity-related sleep disorders, ISRCTN77172005). Results regarding the comparisons between exercise and control groups have been reported in an earlier publication [16]. In the present paper, we focus solely on the comparison between diet and control groups. However, to give an overall picture of the study, baseline characteristics and sleep outcomes at baseline and six months across the three groups are summarized in Tables S1 and S2. The study was approved by the Ethics Committee of the Central Finland Health Care District (7/2011). Informed consent was obtained from all participants prior to the baseline measurements and a copy of the signed consent form was archived.

### 2.1. Participants

**Figure 1:**
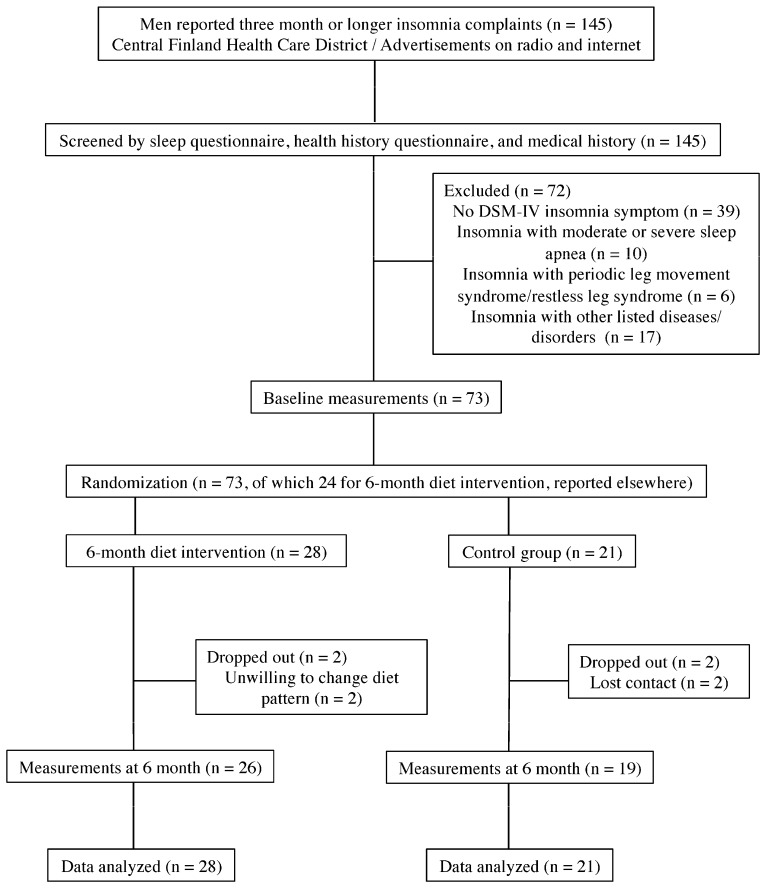
Participant flow of the study.

Participants were 49 Finnish men aged 30–65 years with chronic (three months or longer) complaints of insomnia symptoms. Ninety-four percent (*n* = 46) of them had BMI ≥ 25 kg/m^2^. Participants were voluntarily recruited through the outpatient clinics and public health care centers in the Central Finland Health Care District, or through advertising on the local radio news media and the Internet. The summary of participant flow is presented in Figure 1.

The modified Basic Nordic sleep questionnaire (BNSQ) [17], the health and behavior questionnaire, and participant’s medical history were collected and reviewed by a physician for screening. Insomnia symptoms were classified according to the DSM-IV-TR criteria (without the criterion of daytime consequences) from answers to the modified BNSQ. Individuals were considered to have chronic insomnia symptoms if one or more of the following symptoms had occurred at least three nights per week, during the past three months: (1) Difficulty initiating sleep (subjective SOL ≥ 30 min); (2) Difficulty maintaining sleep (awakening during sleep ≥3 times/night, or difficulty in falling asleep after nocturnal awakening with total wake after sleep onset ≥30 min); (3) Early morning awakenings (wake up ≥30 min earlier than desired in the morning and unable to fall asleep again); (4) Non-restorative sleep [18,19].

Exclusion criteria were: (1) other sleep disorders include moderate or severe apnea (Apnea-hypopnea index, AHI ≥ 15), restless leg syndrome and periodic leg movement disorder (periodic leg movement arousal index > 15), narcolepsy, REM behavior disorder, and circadian rhythm disorder; (2) Medical history during the past three years related to diseases such as cardiovascular disease, heart failure, liver disease, and cancer; (3) Current diagnosis of major depression; (4) History of other major mental illness or substance abuse; (5) History of cognitive impairment and major neurological disorders; (6) History of eating disorders; (7) Taking special diet at the moment; (8) Chronic pain conditions; (9) Regular use of sedatives, hypnotics, and painkillers; (10) Shift work [12].

### 2.2. Measurements

All measurements were carried out before randomization, and after the six-month intervention period. In addition, nutrient intake and anthropometry were measured at three months.

#### 2.2.1. Descriptive Characteristics

Age, education, employment, and smoking habits were elicited at baseline with the health and behavior questionnaire. Age of onset of insomnia and occurrences of insomnia symptoms were elicited with the baseline modified BNSQ.

#### 2.2.2. Energy Consumption and Nutrients Intake

A three-day diet diary (two weekdays and one weekend) collected the type, item, and estimated portion of all food and drink intake during each day. Archiving of diet information, calculation of nutrients intake, total calories, and proportions of energy-yielding nutrients in total calories (E%) were carried out by the Micro-Nutrica software (The Social Insurance Institution of Finland, Turku, Finland).

#### 2.2.3. Anthropometry and Fat Mass

All anthropometric measurements were performed after overnight fasting (12 h). Height was measured to the nearest 0.5 cm using a fixed wall scale. Weight was determined to the nearest 0.1 kg using a calibrated physician weight scale. BMI was calculated as weight (kg) per height^2^ (m^2^). Neck, chest, waist, and hip circumferences were determined to the nearest 0.1 cm by a measuring tape using standardized procedures, and the average value of three measurements was taken for analysis. Blood pressure were measured using an oscillometric monitor in sitting position after five-minute resting, average value of three measurements was retained. Fat mass was determined using dual energy X-ray densitometry (DXA; Prodigy, GE Lunar, Madison, WI, USA).

#### 2.2.4. Energy Expenditures

A seven-day physical activity diary was collected on the same days as sleep measurements. The diary recorded primary living activity at 30-min intervals over 24 h. Energy expenditures were calculated as metabolic equivalent multiplied by minutes per day (MET min/day), according to the 2011 Compendium of Physical Activities [20]. Expenditures were categorized into exercise and recreational activity (e.g., walking a dog, berry picking), livelihood physical activity (e.g., personal care, housework, commuting, occupational activities), as well as sedentary behaviors (METs ≤ 1.5 while in a sitting or reclining posture) and sleep [21].

#### 2.2.5. Objective Sleep Measurement

Home-based objective sleep data were collected by an unobtrusive online sleep monitoring system (Beddit pro; Beddit Ltd., Espoo, Finland). The system included a piezoelectric bed sensor. Ballistocardiographic signals were sampled by the piezoelectric sensor at 140 Hz and simultaneously uploaded to a web server through the Internet, where sleep/wake status was classified in 30-s epochs based on heart rate variability, respiration rate variability, and binary actigram [22]. An ambient brightness sensor, included in the system, was placed in the bedroom for determining lights-out time. For participants who had a bed partner, sensor attachment was considered to avoid overlapping measurements. Participants were instructed to mention conditions that might have affected the measurements, such as children and pets in the bedroom, in the sleep diary. Measurement was set automatically to start each evening at 18:00, and end at noon the next day. Total sleep time (TST), SOL (determined as the duration from being present in bed with lights out to the first five minutes of consecutive sleep) [23], wakefulness after sleep onset (WASO), and sleep efficiency (SE) were obtained for each night. Sleep was measured for seven nights, including two weekends. Measurements were taken within 14 days both before and after the six-month study period. For analyses, average values across the nights were used; at least five nights’ valid data at both baseline and six months were needed. Validation of the sleep/wake in 30-s epochs was carried out against two-night polysomnography measurement (31 subjects with insomnia complaints, age (±SD) = 51.8 ± 8.4 years, BMI = 30.9 ± 4.8 kg/m^2^). Correlations in sleep outcomes were obtained as follow: TST (Pearson’s *r* = 0.85, *p* < 0.001), SOL (Pearson’s *r* = 0.81, *p* < 0.001), WASO (Kendall’s tau-b = 0.74, *p* < 0.001), SE (Kendall’s tau-b = 0.68, *p* < 0.001) [16].

#### 2.2.6. Sleep Diary and Modified BNSQ

The seven-night sleep diary was collected on same nights with objective sleep measurement. Items included time of going to bed, estimated time of falling asleep, number of nocturnal awakenings, final waking-up time, morning-rated subjective sleep quality, fatigue upon awakening, nap duration, and other issues related with sleep. The average values for the recorded nights were used for analyses. Epworth sleepiness scale (ESS) score [24], Rimon’s brief depression scale score [25], insomnia symptom frequency, and other subjective sleep assessment results were elicited by the modified BNSQ.

### 2.3. Randomization

After the baseline measurements, participants were randomized and allocated into the diet intervention or control group, by an external statistician. Randomization was stratified by age and BMI (≤ or >medians) with a block size of 5, using SAS v. 9.2, (SAS Institute, Cary, NC, USA).

### 2.4. Interactive Diet Intervention

A six-month individualized diet intervention program was made according to the three-day diet diary results and BMI at baseline. Individualized programs were introduced to each participant face-to-face by study nutritionists on the first day of intervention. Diet suggestions were made according to the Finnish Nutrition Recommendations [26]. Suggested proportions of energy-yielding nutrients were: 40%–45% of carbohydrate in total daily energy intake (E%) with <5 E% sucrose; 35–40 E% total fat with ≤10 E% saturated fatty acids (SFA), 15–20 E% monounsaturated fatty acids (MUFA), and 5–10 E% polyunsaturated fatty acids (PUFA); and 20 E% protein [26]. In addition, greater consumption of dietary fiber, vitamin A, vitamin D, vitamin E, B vitamins, vitamin C, magnesium, and potassium was recommended through selected food options (cereals, vegetables, fruits, berries, nuts, legumes, mushrooms, etc.). Participants with overweight and obesity (*n* = 27) were advised to gradually reduce their daily energy intake by 300–500 kcal during the first three months, with a target of reducing body weight by 3 kg. After this period, calorie intake was suggested to remain at the reduced level. Two intermediate face-to-face counseling sessions were held in the first and the fourth month of the intervention. Each intermediate session involved individualized diet counseling with a nutritionist, and a cooking course in which examples of meals that fulfilled the nutritional criteria of this study were introduced.

During the intervention, an online diet and nutrition counseling service (MealTracker, Wellness Foundry Holding Ltd., Helsinki, Finland) was utilized for supervising individuals’ dietary intake and providing diet suggestions. Participants were instructed to photograph all daily dietary intakes (including drinks) using a smartphone or a digital camera, and upload all photos to the server 1–3 days per week during the intervention. The photos were uploaded via a mobile application, or through the service’s website. According to the uploaded photos, a nutritionist assessed each individual’s daily calorie intake and consumptions of nutrients. Individualized feedback including dietary intake facts and instructions for diet adjustment in the upcoming days was thus formulated and sent to participants via mobile text message and e-mail each day with uploaded information. Diet photos were saved in each participant’s account in the server, which was only accessible to the participant and the nutritionist. A training session for taking and uploading diet photos was held prior to the intervention. All participants in the diet group were able to use the service correctly.

### 2.5. Control Group

Controls were instructed to keep their habitual, pre-recruitment lifestyle for six months. They were given an opportunity to participate in the diet plus exercise intervention program for three months after the study period.

### 2.6. Statistical Analysis

The estimated change of the objective SOL was based on published data [16,27]. Statistical power was over 80% to detect a 30% lowered SOL in the diet group from baseline, and no change of SOL in the control group, with the unbalanced allocation of 28 and 21 participants in each randomized group.

Analyses were carried out following the intention-to-treat principle. For participants with missing or incomplete values at follow-ups, the last observed values were carried forward. All analyses were performed using IBM SPSS statistics version 20 (SPSS, Inc., Chicago, IL, USA). All tests were two-tailed; a *p* value less than 0.05 was set as significant. The Shapiro–Wilk W test and Levene’s test were used to examine the normality and homogeneity, respectively. Skewed data were transformed by natural logarithm. Baseline differences between groups were evaluated by one-way analysis of variance (ANOVA), or Pearson’s χ^2^ test. Time-by-group differences were evaluated by analysis of covariance (ANCOVA), controlling for the baseline values. Within-group differences were evaluated by repeated measures ANOVA, followed with Bonferroni corrections for multiple comparisons. In addition, Pearson’s correlation coefficients were calculated between changes from baseline to six months for selected variables.

## 3. Results

Baseline descriptive characteristics by group are given in Table 1. Retention rates between diet and control groups were comparable (Diet = 26/28, Control = 19/21, *p* = 0.579, Fisher’s exact test).

### 3.1. Compliance with Diet Interventions

On average, participants in the diet group who attended the six-month follow-up measurements uploaded 1.9 ± 1.1 (SD) days per week during intervention. All of these participants attended all three counseling sessions. Two participants dropped out during the first month of the study, both due to the unwillingness to change diet. There were no diet photos uploaded from the participants who dropped out.

### 3.2. Energy Consumption and Nutrient Intake

**Figure 2:**
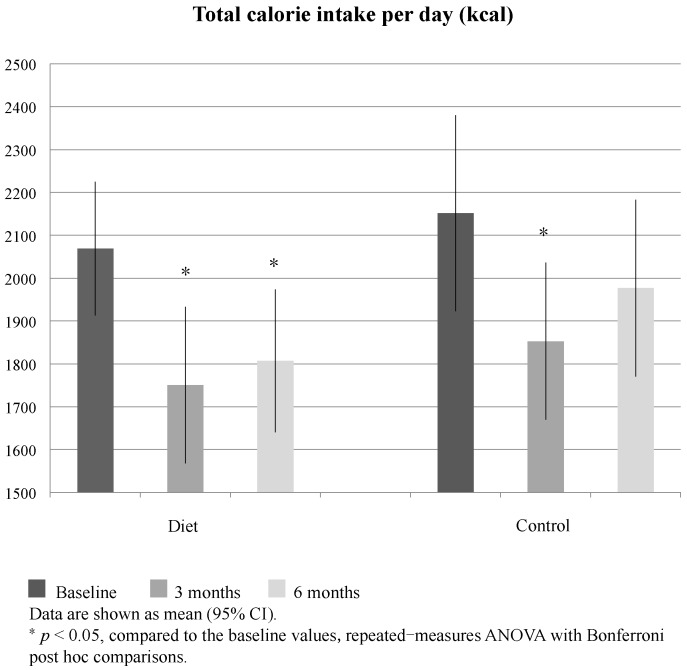
Daily calorie intake by three-day diet diary at baseline vs. three and six months.

Within the diet group, total energy intake was reduced at six months compared to baseline (*p* = 0.006, Figure 2); however, changes in other nutrients were not detected. Total energy intake was reduced at three months in both groups (*p* = 0.001 and 0.012, respectively). Proportions of energy-yielding nutrients in total calories did not show significant change in either group. Compared to the controls, the diet group had greater intakes of potassium (2158 vs. 1806 mg/1000 kcal, *p* = 0.029, ANCOVA controlling for baseline) and magnesium (219 vs. 193 mg/1000 kcal, *p* = 0.036, ANCOVA controlling for baseline) at three months (not shown in figure).

### 3.3. Anthropometry, Fat Mass, and Energy Expenditures

Body weight, total fat mass, and waist circumference decreased significantly in the diet group compared to the control group (*p* = 0.043 to 0.009, Table 2). No significant changes in physical activity, sedentary time, or total energy expenditure were found in either group during the intervention.

### 3.4. Objective Sleep Parameters

**Figure 3:**
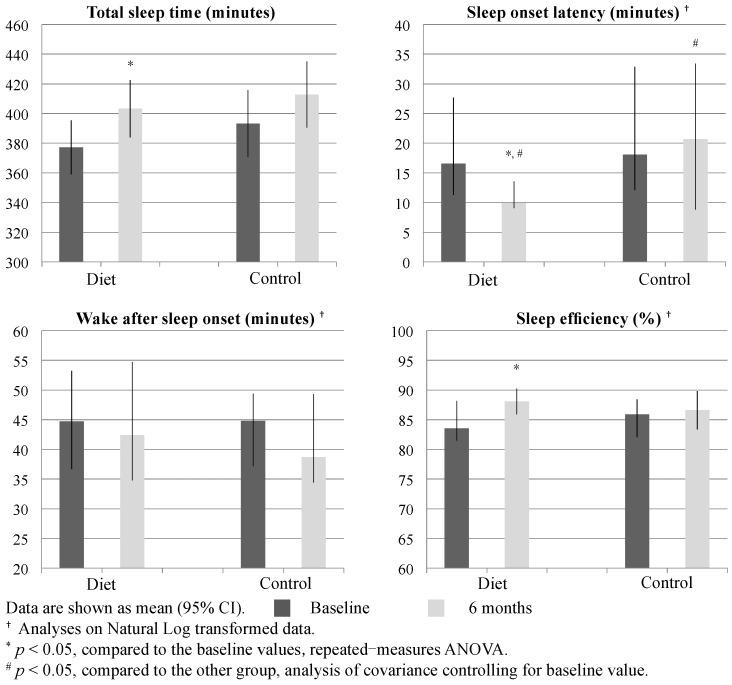
Sleep outcomes by piezoelectric system at baseline and six months.

At baseline, the valid objective sleep data for analyzing were on average 6.4 ± 1.1 and 6.8 ± 0.6 nights in diet and control groups, respectively. At six months, the corresponding numbers were 6.4 ± 0.9 and 6.6 ± 0.7. There was no between-group difference in the number of nights analyzed at baseline or six months (*p* = 0.110 and 0.613, respectively, one-way ANOVA). Nights marked by participants as being subject to significant disturbance in the measurement process (such as a pet sleeping in the bed) were excluded. Results of the objective sleep measurements are given in Figure 3. Compared to the control group, the diet group showed shorter SOL (*p* < 0.001) after intervention. Within the diet group, prolonged TST (*p* = 0.004), curtailed SOL (*p* < 0.001), and increased sleep efficiency were found (*p* = 0.004) through intervention. In the diet group, change of objective SOL through intervention did not correlate with changes in body weight or fat mass (both *p* > 0.05, Pearson’s *r*).

### 3.5. Subjective Sleep Assessments

No time-by-group difference was detected among subjective sleep parameters (Table 3). Within the diet group, nocturnal awakenings (*p* = 0.035), number of nocturia (*p* = 0.001), and Rimon’s depression score (*p* = 0.029) were reduced compared to the baseline values.

## 4. Discussion

In this study we found that a six-month diet intervention was associated with reduced body weight, fat mass, and curtailed objective sleep onset latency among overweight and obese men with chronic insomnia symptoms.

Previous studies have investigated whether adjusting total energy intake may alter sleep parameters. A recent study reported that one-week ad libitum feeding with increased calorie density (high fat diet) resulted in increased sleep/wake fragmentation in mice [11], which indicates a link between excessive energy consumption and disturbed sleep. A human study of acute diet alteration in normal sleepers has shown that, compared to limited total energy intake, sleep after ad libitum feeding was characterized by longer SOL and shorter slow wave sleep [10]. By suggesting controlled total calorie intake for a period of six months, our study further infers the association between energy restriction and improved nocturnal sleep parameters such as SOL in individuals with insomnia symptoms.

Nonetheless, it is not known whether total energy intake or the relative proportion of energy-yielding nutrients plays the more important role in mediating the effect of diet on sleep. In this study, subjects who received diet intervention reported significantly reduced total energy intake. However the proportions of the major categories of energy-yielding nutrients (carbohydrates, fat, protein) were not significantly changed through intervention. Hence energy reduction per se with negative energy balance may contribute to faster sleep onset among overweight individuals. More studies are needed in order to identify whether altering energy proportions without changing the total calories in the diet has the same effect on sleep among humans. So far, only a few studies have tested the effects of isocaloric diets with different nutrient composition on sleep and sleepiness. These studies suggested an association between macronutrient proportion and sleep-related parameters, but the results are inconclusive due to the small sample size and the non-randomized study nature. A study dating back to 1975 found less slow wave sleep following a two-day high-carb-low-fat diet than after a two-day low-carb-high-fat diet [28]. Another study compared the acute effect of high-carb/low-fat and low-carb/high-fat diets on sleepiness, and found a stronger feeling of sleepiness at 2–3 h following the former dietary pattern [29].

The mechanisms underlying the association between reduced energy intake and improved nocturnal sleep parameters remain to be revealed. It is known that diet among overweight and obese individuals is characterized by a larger proportion of fat in total energy consumption [12]. One study has shown that chronic high fat feeding reduces prepro-orexin level in hypothalamus in obese mice [30]. Since orexin is an important neuropepitide for maintaining wakefulness and regulating energy balance, we hypothesize that by reducing total energy intake, orexin signaling in the hypothalamus is strengthened, which induces more stable wakefulness and less sleep duration during the daytime. Less daytime sleep may further contribute to better nocturnal sleep, with higher sleep efficiency [31,32]. In addition, orexin neurons showed impaired thermo-sensitivity after high calorie density feeding [33], which prolonged sleep onset according to the mechanism that thermo downregulation triggers sleep. Therefore, we assume that lowering calorie intake may also recover the blunted thermo response of orexin neurons.

In the present study, we found that subjects in the diet intervention group had a greater intake of potassium and magnesium than the controls. This was partially in line with earlier reports that intakes of non-energy-yielding nutrients such as fiber, Vitamin D, potassium, and magnesium are associated with sleep [10,13,34,35,36]. Although the mechanisms regarding the role of potassium and magnesium on sleep regulation are not yet understood, these results suggest we should pay attention to micronutrients that may affect sleep.

Another noteworthy point in this study is that diet intervention led to lower body weight, fat mass, and waist circumference, and such contrasts were not just the result of weight and fat mass reductions in the diet group, but also, more significantly, of increased weight and fat mass among the controls. These results, together with other evidence, indicate that chronic insomnia symptoms contribute to weight gain [37,38,39,40,41,42,43]. This process is possibly caused by diet-induced positive energy balance. We also tested whether diet-induced weight change was associated with changes in objective sleep parameters; however, no significant correlation was found among dieters in our study. This might either be due to the small sample size (*n* = 28 in the diet group) or to the fact that the amount of body weight alteration is not necessarily related to absolute changes in objective sleep parameters.

The present study was subject to several limitations. First, apart from the objective sleep onset latency, no time-by-treatment difference was observed in the sleep parameters. Thus, in comparison with other non-pharmaceutical methods for mitigating insomnia symptoms, such as cognitive behavioral treatment and aerobic exercise [16,23,27,44], diet intervention may have a weaker treatment efficacy for insomnia. Nevertheless, as the average weight reduction did not achieve the goal (3 kg) under such a diet intervention protocol, stricter calorie intake control should be introduced in order to test the effects of diet on sleep in this population. Moreover, diet information was only recorded at baseline, three months, and six months, which limited information on actual nutrient intake during intervention. Finally, we did not exclude participants with mild sleep apnea (5 ≤ AHI < 15) due to the high prevalence of this symptom among overweight and obese men (up to 61.4%) [45]. Hence, it is possible that the improvement in sleep was partially due to improvements in sleep breathing.

To our knowledge, this is the first randomized controlled study investigating the effects of diet intervention on sleep among individuals with insomnia symptoms. The results suggest that a six-month diet intervention with reduced energy intake and recommended nutrient composition reduces objective SOL among overweight and obese men with chronic insomnia symptoms. The findings of the present study provide new evidence for the potential role of dietary interventions in overweight and obese men with insomnia.

## 5. Conclusions

Modest energy restriction and optimized nutrient composition shorten sleep onset latency in overweight and obese men with insomnia symptoms.

## Figures and Tables

**Table 1:** Descriptive characteristics at baseline.

	Diet (*n* = 28)	Control (*n* = 21)	*p* ^#^
	Mean (95% CI)	Mean (95% CI)	
Age (year)	51.0 (47.3 to 54.8)	52.6 (48.0 to 57.2)	0.592
Age when insomnia complaint started (year)	37.4 (33.1 to 41.6)	39.8 (33.7 to 46.0)	0.482
Height (cm)	178.9 (177.0 to 180.8)	178.3 (175.6 to 180.9)	0.696
Weight (kg)	93.8 (89.2 to 98.4)	93.1 (85.2 to 100.9)	0.860
BMI (kg/m^2^)	29.4 (27.9 to 30.8)	29.2 (27.2 to 31.2)	0.879
Systolic blood pressure (mmHg)	142.8 (139.0 to 146.6)	140.7 (135.2 to 146.3)	0.513
Diastolic blood pressure (mmHg)	88.8 (84.9 to 92.6)	91.4 (86.7 to 96.1)	0.363
**Occurrences**	**Percentage**	**Percentage**	
Difficulty initiating sleep	42.9	42.9	1.000
Difficulty maintaining sleep	57.1	76.2	0.166
Early morning awakenings	32.1	23.8	0.523
Non-restorative sleep	39.3	42.9	0.801
Smoking presently	14.3	19.0	0.655
At least tertiary degree education	82.1	95.2	0.166
Employed	82.1	71.4	0.374

^#^ One-way ANOVA or Pearson’s χ^2^ test.

**Table 2:** Anthropometry, fat mass, and energy expenditures at baseline and follow-ups.

	Diet			Control		
	Baseline	3 Months	6 Months	Baseline	3 Months	6 Months
**Anthropometry**						
Weight (kg)	93.8 (89.2 to 98.4)	92.7 (88.3 to 97.0) ^#^	92.7 (88.1 to 97.4) ^#^	93.1 (85.2 to 100.9)	93.5 (85.5 to 101.5) ^#^	94.4 (86.3 to 102.5) *^,#^
Neck circumference (cm)	42.0 (41.0 to 43.0)	41.9 (40.9 to 43.0)	42.2 (41.3 to 43.2)	41.9 (40.6 to 43.3)	42.0 (40.5 to 43.4)	42.6 (41.2 to 44.0) *
Chest circumference (cm)	109.4 (106.8 to 112.0)	109.2 (106.5 to 111.9)	109.7 (106.9 to 112.5)	107.7 (102.6 to 112.9)	108.0 (102.9 to 113.1)	109.5 (104.5 to 114.5) *
Waist circumference (cm)	106.6 (102.9 to 110.2)	106.1 (102.7 to 109.4)	105.9 (102.4 to 109.4) ^#^	105.0 (99.9 to 110.1)	105.4 (99.9 to 110.8)	106.7 (101.3 to 112.2) *^,#^
Hip circumference (cm)	104.5 (101.9 to 107.1)	103.1 (100.1 to 106.1)	103.5 (100.4 to 106.7)	102.7 (98.6 to 106.7)	103.5 (99.6 to 107.3)	104.0 (100.0 to 108.1)
**Fat mass**						
Total fat mass (kg)	27.5 (24.2 to 30.7)	n/a	26.8 (23.5 to 30.2) ^#^	28.0 (23.6 to 32.5)	n/a	28.9 (24.0 to 33.8) *^,#^
Trunk fat mass (kg)	17.6 (15.6 to 19.6)	n/a	17.2 (15.1 to 19.3)	17.7 (14.5 to 20.8)	n/a	18.2 (14.9 to 21.5) *
**Energy expenditures**						
Total expenditure (MET min/day)	2346.1 (2254.9 to 2437.3)	n/a	2398.8 (2299.7 to 2498.0)	2341.1 (2224.0 to 2458.1)	n/a	2322.6 (2210.0 to 2435.3)
Exercise and recreational physical activity (MET min/day)	226.7 (150.8 to 302.6)	n/a	292.5 (194.5 to 390.4)	249.5 (145.2 to 353.8)	n/a	254.3 (151.9 to 356.7)
Household physical activity (MET min/day)	840.9 (675.4 to 1006.5)	n/a	845.4 (689.9 to 1000.9)	803.0 (631.5 to 974.5)	n/a	751.6 (548.6 to 954.6)
Sedentary behaviors (MET min/day)	846.7 (739.0 to 954.4)	n/a	801.7 (706.1 to 897.3)	824.6 (737.1 to 912.1)	n/a	845.0 (758.3 to 931.7)

Data are shown as mean (95% CI); ^#^ *p* < 0.05, compared to the other group, analyses of covariance controlling for baseline values; * *p* < 0.05, compared to the baseline values, repeated measures ANOVA with Bonferroni post hoc comparisons.

**Table 3:** Sleep outcomes by sleep diary and sleep questionnaire at baseline and six months.

	Diet			Control			Time by Group
	Baseline	6 Months	*p*	Baseline	6 Months	*p*	*p* ^#^
**Sleep diary**							
Sleep onset latency (min) ^†^	21.0 (13.5 to 31.0)	20.0 (13.3 to 23.8)	0.122	21.5 (17.3 to 41.8)	25.0 (15.0 to 42.5)	0.463	0.255
Nocturnal awakenings (numbers/night)	2.3 (1.8 to 2.9)	1.8 (1.3 to 2.4)	0.035	2.6 (1.8 to 3.4)	2.3 (1.8 to 2.8)	0.293	0.305
Nocturia (times/night)	0.8 (0.6 to 1.1)	0.5 (0.3 to 0.6)	0.001	0.7 (0.4 to 0.9)	0.5 (0.3 to 0.8)	0.080	0.075
Morning-rated sleep quality (1–4) ^a^	2.4 (2.1 to 2.7)	2.7 (2.4 to 2.9)	0.094	2.4 (2.2 to 2.5)	2.4 (2.2 to 2.6)	0.785	0.153
Fatigue upon awakening (1–4) ^b^	2.2 (2.0 to 2.4)	2.0 (1.7 to 2.2)	0.062	1.9 (1.6 to 2.1)	2.0 (1.8 to 2.2)	0.300	0.292
Nap (min/day) *	17.1 (11.2 to 23.1)	14.0 (8.1 to 19.9)	0.388	12.5 (4.4 to 20.6)	11.9 (2.2 to 21.6)	0.885	0.957
**Sleep questionnaire**							
Difficulty initiating sleep (1–5) ^c^	2.5 (2.0 to 3.0)	2.3 (1.9 to 2.7)	0.227	2.8 (2.2 to 3.3)	2.7 (2.1 to 3.2)	0.540	0.376
Early morning awakenings (1–5) ^c^	3.0 (2.5 to 3.5)	2.8 (2.2 to 3.3)	0.246	3.0 (2.5 to 3.5)	3.1 (2.5 to 3.7)	0.452	0.182
Sleep less than 5 h in last month (1–6) ^d^	2.9 (2.4 to 3.3)	2.6 (2.1 to 3.1)	0.355	3.0 (2.3 to 3.6)	2.8 (2.2 to 3.3)	0.384	0.718
Habitual sleep duration (h)	6.6 (6.1 to 7.1)	6.7 (6.2 to 7.2)	0.706	6.7 (6.2 to 7.2)	6.6 (6.1 to 7.1)	0.545	0.658
Desired sleep duration (h)	7.9 (7.5 to 8.4)	7.8 (7.3 to 8.3)	0.398	8.1 (7.6 to 8.5)	7.9 (7.5 to 8.3)	0.117	0.776
Epworth sleepiness scale score	6.6 (5.2 to 8.0)	6.3 (4.9 to 7.7)	0.612	8.3 (6.2 to 10.5)	7.4 (5.2 to 9.7)	0.056	0.679
Rimon’s depression score ^†^	5.0 (4.0 to 7.0)	4.0 (1.3 to 6.0)	0.029	4.0 (3.0 to 7.5)	3.0 (2.5 to 5.5)	0.187	0.358

Data are shown as mean (95% CI) unless specified otherwise; ^†^ Comparisons under Natural Log transformed data, values are shown as the medians and 25th through 75th percentiles; ^#^ Analyses of covariance controlling for baseline values; * Diet (*n* = 19), Control (*n* = 12); ^a^ 1 = Very poor; 2 = Quite poor; 3 = Good; 4 = Very good; ^b^ 1 = Not fatigued at all; 2 = A little fatigued; 3 = Quite fatigued; 4 = Very fatigued; ^c^ 1 = Never/less than once per month; 2 = Less than once per week; 3 = 1–2 days per week; 4 = 3–5 days per week; 5 = daily or almost daily; ^d^ 1 = 0 night; 2 = 1–5 nights; 3 = 6–10 nights; 4 = 11–15 nights; 5 = 16–20 nights; 6 = more than 20 nights.

## Supplementary Materials

The following are available online at http://www.mdpi.com/2072-6643/8/11/751/s1, Table S1: Descriptive characteristics of three groups at baseline; Table S2: Sleep outcomes of three groups at baseline and six months.
