# Supplementary material for: Effect of Six-Month Diet Intervention on Sleep among Overweight and Obese Men with Chronic Insomnia Symptoms: A Randomized Controlled Trial
Source: Nutrients. 2016 Nov 23;8(11):751. doi: 10.3390/nu8110751 (PMC5133133; doi:10.3390/nu8110751)
Supplement: Supplementary file 1 [file nutrients-08-00751-s001.docx]

Supplementary Materials: Effect of Six-Month Diet Intervention on Sleep among Overweight and Obese Men with Chronic Insomnia Symptoms: A Randomized Controlled Trial

Xiao Tan, Markku Alén, Kun Wang, Jarkko Tenhunen, Petri Wiklund, Markku Partinen and Sulin Cheng

**Table S1.** Descriptive characteristics of three groups at baseline.

|  | **Exercise (*n* = 24)** | **Diet (*n* = 28)** | **Control (*n* = 21)** |
| --- | --- | --- | --- |
|  | **Mean (95% CI)** | **Mean (95% CI)** | **Mean (95% CI)** |
| Age (year) | 51.2 (46.6 to 55.8) | 51.0 (47.3 to 54.8) | 52.6 (48.0 to 57.2) |
| Age when insomnia complaint started (year) | 40.9 (35.5 to 46.3) | 37.4 (33.1 to 41.6) | 39.8 (33.7 to 46.0) |
| Height (cm) | 177.4 (174.7 to 180.1) | 178.9 (177.0 to 180.8) | 178.3 (175.6 to 180.9) |
| Weight (kg) | 92.3 (86.2 to 98.5) | 93.8 (89.2 to 98.4) | 93.1 (85.2 to 100.9) |
| BMI (kg/m^2^) | 29.3 (27.6 to 31.0) | 29.4 (27.9 to 30.8) | 29.2 (27.2 to 31.2) |
| Systolic blood pressure (mmHg) | 140.0 (132.4 to 147.7) | 142.8 (139.0 to 146.6) | 140.7 (135.2 to 146.3) |
| Diastolic blood pressure (mmHg) | 90.0 (85.7 to 94.4) | 88.8 (84.9 to 92.6) | 91.4 (86.7 to 96.1) |
| Occurrences | Percentage | Percentage | Percentage |
| Difficulty initiating sleep | 50.0 | 42.9 | 42.9 |
| Difficulty maintaining sleep | 54.2 | 57.1 | 76.2 |
| Early morning awakenings | 33.3 | 32.1 | 23.8 |
| Non-restorative sleep | 50.0 | 39.3 | 42.9 |
| Smoking presently | 29.2 | 14.3 | 19.0 |
| At least tertiary degree education | 91.7 | 82.1 | 95.2 |
| Employed | 82.6 | 82.1 | 71.4 |

No between-group differences were detected by one-way ANOVA or Pearson’s χ^2^ test.

**Table S2.** Sleep outcomes of three groups at baseline and six months.

|  | **Exercise** | | **Diet** | | **Control** | | ***p* ^#^** | |
| --- | --- | --- | --- | --- | --- | --- | --- | --- |
|  | **Baseline** | **6 Months** | **Baseline** | **6 Months** | **Baseline** | **6 Months** | **Exercise Control** | **Diet Control** |
| Piezoelectric system | | | | | | | | |
| TST (min) | 376.8 (353.7 to 399.8) | 390.1 (364.8 to 415.3) | 377.2 (359.0 to 395.4) | 403.3 (384.1 to 422.6) * | 393.3 (370.9 to 415.8) | 412.8 (390.4 to 435.2) | 0.331 | 0.995 |
| SOL (min) ^†^ | 23.9 (14.0 to 42.2) | 13.7 (6.7 to 22.6) * | 16.6 (11.3 to 27.7) | 9.9 (9.1 to 13.6) * | 18.1 (12.1 to 32.9) | 20.7 (8.8 to 33.4) | 0.009 | 0.001 |
| WASO (min) ^†^ | 48.3 (43.9 to 62.2) | 39.1 (35.9 to 52.3) * | 44.7 (36.7 to 53.2) | 42.4 (34.8 to 54.7) | 44.8 (37.2 to 49.4) | 38.7 (34.4 to 49.3) | 0.163 | 0.798 |
| SE (%) ^†^ | 81.7 (78.3 to 86.7) | 87.1 (83.1 to 89.3) * | 83.5 (81.5 to 88.2) | 88.1 (85.9 to 90.2) * | 85.9 (82.1 to 88.4) | 86.6 (83.4 to 89.8) | 0.164 | 0.135 |
| Sleep diary | | | | | | | | |
| SOL (min) ^†^ | 30.0 (12.0 to 60.0) | 21.0 (12.0 to 40.0) | 21.0 (13.5 to 31.0) | 20.0 (13.3 to 23.8) | 21.5 (17.3 to 41.8) | 25.0 (15.0 to 42.5) | 0.461 | 0.288 |
| Nocturnal awakenings (numbers/night) | 2.6 (1.8 to 3.3) | 1.9 (1.4 to 2.4) * | 2.3 (1.8 to 2.9) | 1.8 (1.3 to 2.4) * | 2.6 (1.8 to 3.4) | 2.3 (1.8 to 2.8) | 0.183 | 0.256 |
| Nocturia (times/night) | 0.8 (0.5 to 1.1) | 0.7 (0.4 to 1.0) | 0.8 (0.6 to 1.1) | 0.5 (0.3 to 0.6) * | 0.7 (0.4 to 0.9) | 0.5 (0.3 to 0.8) | 0.624 | 0.084 |
| Morning-rated sleep quality (1–4) ^a^ | 2.5 (2.3 to 2.7) | 2.7 (2.5 to 2.9) * | 2.4 (2.1 to 2.7) | 2.7 (2.4 to 2.9) | 2.4 (2.2 to 2.5) | 2.4 (2.2 to 2.6) | 0.112 | 0.148 |
| Fatigue upon awakening (1–4) ^b^ | 2.2 (2.0 to 2.4) | 1.9 (1.7 to 2.1) * | 2.2 (2.0 to 2.4) | 2.0 (1.7 to 2.2) | 1.9 (1.6 to 2.1) | 2.0 (1.8 to 2.2) | 0.203 | 0.368 |
| Sleep questionnaire | | | | | | | | |
| Difficulty initiating sleep (1–5) ^c^ | 2.8 (2.2 to 3.3) | 2.2 (1.7 to 2.6) * | 2.5 (2.0 to 3.0) | 2.3 (1.9 to 2.7) | 2.8 (2.2 to 3.3) | 2.7 (2.1 to 3.2) | 0.028 | 0.343 |
| Early morning awakenings (1–5) ^c^ | 2.7 (2.2 to 3.3) | 2.6 (2.1 to 3.1) | 3.0 (2.5 to 3.5) | 2.8 (2.2 to 3.3) | 3.0 (2.5 to 3.5) | 3.1 (2.5 to 3.7) | 0.238 | 0.191 |
| Epworth sleepiness scale score | 8.4 (7.0 to 9.8) | 6.8 (5.5 to 8.1) * | 6.6 (5.2 to 8.0) | 6.3 (4.9 to 7.7) | 8.3 (6.2 to 10.5) | 7.4 (5.2 to 9.7) | 0.375 | 0.783 |
| Rimon’s depression score ^†^ | 4.0 (2.0 to 7.8) | 2.5 (0.0 to 4.0) * | 5.0 (4.0 to 7.0) | 4.0 (1.3 to 6.0) * | 4.0 (3.0 to 7.5) | 3.0 (2.5 to 5.5) | 0.067 | 0.337 |

TST: Total sleep time; SOL: Sleep onset latency; WASO: Wakefulness after sleep onset; SE: Sleep efficiency; Data are shown as Mean (95% CI) unless further notified; ^#^ Time by group comparisons, analyses of covariance controlling for baseline values with Tukey least significant difference post hoc tests; * *p* < 0.05, compared to the baseline value, repeated measures analyses of variance; ^†^ Comparisons under Natural Log transformed data, values are shown as the medians and 25th through 75th percentiles; ^a^ 1 = Very poor; 2 = Quite poor; 3 = Good; 4 = Very good; ^b^ 1 = Not fatigued at all; 2 = A little fatigued; 3 = Quite fatigued; 4 = Very fatigued; ^c^ 1 = Never/less than once per month; 2 = Less than once per week; 3 = 1–2 days per week; 4 = 3–5 days per week; 5 = daily or almost daily.
